# The Health of Doctors

**Published:** 1990-03

**Authors:** M. G. Wilson


### Book Review

**THE HEALTH OF DOCTORS**
**Clive Richards**
King Edwards Hospital Fund for London £2.50

Dr Richards has chosen a difficult and important subject for his study. He states the known facts immediately, doctors have twice as many deaths from road accidents as the general population, a cirrhosis and suicide rate three times as high and a drug addiction rate at least thirty times higher. Many of these problems are related to stress, heavy work load, high responsibility, fatigue, disruptions from unpredictable emergencies leading to alienation from family and social networks. This burden is inseparable from the life of most doctors and accepted by them. Because it is their common lot they are loth to complain about it or to seek advice from colleagues, they soldier on and treat themselves until they reach breaking point. Dr Richards decided to look at this problem in depth by means of questionnaires sent to GP colleagues, in fact all the GPs in contract with the Avon Family Practioner Committee. The questionnaires were anonymous. The extraordinarily high response (86%) showed how doctors welcomed this opportunity to unburden themselves. The results are closely analysed and with quotations from individual responses make interesting reading. He observes that while doctors are ready to present themselves to colleages with 'medical problems' e.g. lumps or glycosuria, they are most reluctant to seek help for illnesses that have implications of weakness or being unable to cope, in fact those disorders to which the profession are most vulnerable. A doctor will have a natural reluctance to discuss his/her 'weaknesses' with a partner and two thirds of the sample were registered with GPs outside their own practice but some of these complained about the difficulty of getting an appointment and having to wait in queues. He suggests that 'Consideration should be given to the establishment of a special family doctor service' and ends 'Only now are doctors realising that admitting their own humanity may not be so bad after all'.

A problem stated is a problem half solved and the mere reading of this pamphlet will be therapeutic to those doctors in trouble and prophylactic against it for the rest of us.

The work was undertaken as part of an MSc course at the Department of General Practice at the University of Exeter.

M. G. Wilson

